# When MARCH family proteins meet viral infections

**DOI:** 10.1186/s12985-021-01520-4

**Published:** 2021-03-02

**Authors:** Chunfu Zheng, Yan-Dong Tang

**Affiliations:** 1 Department of Immunology, School of Basic Medical Sciences, Fujian Medical University, Fuzhou, China; 2 State Key Laboratory of Veterinary Biotechnology, Harbin Veterinary Research Institute of Chinese Academy of Agricultural Sciences, Harbin, China; 3 Department of Microbiology, Immunology and Infectious Diseases, University of Calgary, Calgary, AB Canada

**Keywords:** MARCH, Antiviral, Proviral, RING finger, E3 ligase

## Abstract

Membrane-associated RING-CH (MARCH) ubiquitin ligases belong to a RING finger domain E3 ligases family. Recent studies have demonstrated that MARCH proteins play critical roles during various viral infections. MARCH proteins can directly antagonize different steps of the viral life cycle and promote individual viral infection. This mini-review will focus on the latest advances of MARCH family proteins' emerging roles during viral infections.

## Introduction

Ubiquitin is a key post-translational modifier in all eukaryotes regulating thousands of proteins and plays important roles in physiological processes [1]. The ubiquitination process requires the cooperation of E1, E2, and E3 ligases. E3 ligases are categorized based on the mechanisms of ubiquitin transfer into RING (really interesting new gene), HECT (homologous to the E6AP carboxyl terminus), and RBR (RING-between RING fingers) families [2]. Membrane-associated RING-CH (MARCH) family proteins are a recently discovered subfamily of the E3 ubiquitin ligases that harbor a catalytic domain C4HC3 cysteine-histidine finger in their N-terminal cytoplasmic tail transmembrane domains that potentially interacts with an E2 enzyme [3]. 11 members have been identified, and their structure is relatively conserved. Most MARCH family proteins have similar structures: with an amino-terminal RING finger domain, also known as the RING-CH domain, followed by zero, two, or more C-terminal transmembrane domains. Nine of the eleven MARCH family members (MARCH1, MARCH2, MARCH3, MARCH4, MARCH5, MARCH6, MARCH8, MARCH9, and MARCH11) contain hydrophobic transmembrane domains. Generally, a typical MARCH protein is characterized by two transmembrane domains, but there are exceptions. MARCH5 consists of 278 amino-acid residues with an amino-terminal RING finger and four carboxy-terminal transmembrane spans [4]. MARCH6 even contains 14 transmembrane helices [5]. MARCH7 and MARCH10 have no predicted transmembrane domains [6, 7].

The MARCH family proteins play important roles in various biological processes, such as the turnover of immune regulatory molecules on the plasma membrane [8]. Latest studies have revealed the essential functions of MARCH proteins in the battle against multiple viral infections. MARCH proteins target viral proteins for polyubiquitination and degradations through distinct pathways. This review summarizes recent progress in understanding MARCH proteins' properties and their emerging roles in regulating viral infection.

### Antiviral MARCH proteins

#### MARCH1/2

**Fig. 1:**
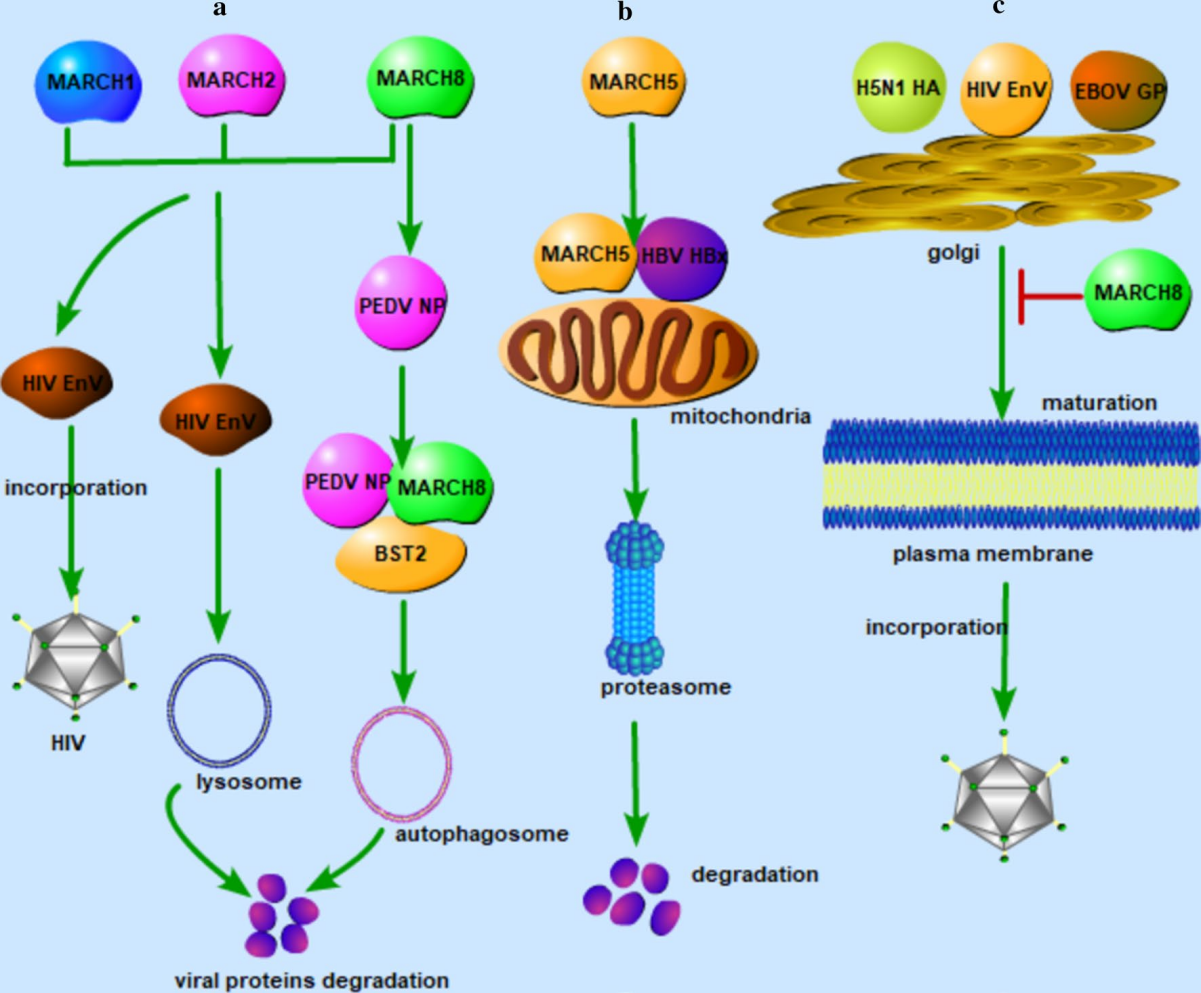
The antiviral functions of MARCH family proteins. **a** MARCH1/2 reduces envelope glycoproteins' incorporation into virions; MARCH1/2/8 target different viral proteins for degradation via lysosome or autophagosome pathway. **b** Mitochondria ubiquitin ligase MARCH5 targets HBV HBX to degradation via the proteasome pathway. **c** MARCH8 inhibits EBOV glycoprotein, HIV-1 envelope glycoprotein, and H5N1 hemagglutinin maturation and virions incorporation. HIV EnV: human immune deficiency virus envelope glycoprotein; PEDV NP: porcine epidemic-diarrhea virus nucleocapsid protein; EBOV GP: ebola virus glycoprotein; HBV HBX: hepatitis B viral × protein; H5N1 HA: influenza virus H5N1 hemagglutinin

MARCH 1 and 2, two proteins of the MARCH family members, are shown to inhibit human immune deficiency virus 1 (HIV-1) infection by reducing envelope glycoproteins' incorporation into virions [9]. Infectivity assays demonstrated that MARCH1/2 suppress viral infection in a dose-dependent manner. Ectopic expression of these proteins in virus-producing cells decreased the effectiveness of viral invasion and down-regulated HIV-1 envelope glycoproteins from the cell surface, resulting in reduced penetration of envelope glycoproteins into virions. MARCH1/2 are demonstrated as antiviral factors, which adds these two proteins to a growing list of host factors inhibiting HIV-1 infection (Fig. 1a). Further investigations are required to study whether these MARCH family members' inhibitory activity against viral envelope glycoproteins could be related to their ability to down-regulate cellular transmembrane proteins, which will enable us to understand the molecular basis of the host defense mechanisms for these proteins.

Interestingly, MARCH2 is shown to be upregulated in HIV-1 infected cells and inhibits HIV-1 production through envelope protein translocation or degradation [10]. MARCH2 inhibits the virions production and infection of HIV-1 through ligase activity-dependent envelope protein degradation and/or intracellular retention, a mechanism shared by MARCH8 that contributes to the inhibition of HIV-1 infection (Fig. 1a). However, unlike MARCH8 and other MARCH proteins whose transcription is not affected during HIV infection, the expression of MARCH2 is substantially upregulated upon HIV-1 infection, giving MARCH2 a unique role in monitoring and regulating the HIV-1 infection-related biological processes.

#### MARCH5

Infection of hepatitis B virus (HBV) promotes chronic liver disease and hepatocellular carcinoma (HCC). The hepatitis B viral x (HBx) protein encoded by the HBV genome contributes to the pathogenesis of HCC. The high expression of MARCH5 in hepatocytes is associated with increased survival of patients with liver cancer since it can sustain cell homeostasis and prevent a malignant tumor. The N-terminal RING domain of MARCH5 is needed to interact with and target HBx for degradation. The lack of E3 ligase activity of MARCH5 mutant could not reduce HBx protein levels. Ectopic expression of HBx increased protein aggregates in semi-denatured detergent agarose gels, and ectopic expression of MARCH5 greatly resolves HBx aggregates via the proteasome-dependent degradation pathway (Fig. 1b). The development of HBx-induced ROS production, mitophagy, and cyclooxygenase-2 gene expression was suppressed when MARCH5 was highly expressed. These findings suggest that MARCH5 is a potential target for ameliorating HBV-mediated liver disease [11].

#### MARCH8

MARCH8 belongs to RING-finger E3 ubiquitin ligases [12, 13]. MARCH8 downregulates many host transmembrane proteins, including major histocompatibility complex (MHC)-II, CD86, interleukin (IL)-1 receptor accessory protein, TNF-related apoptosis-inducing ligand (TRAIL) receptor 1, and the transferrin receptor [14–18]. However, its physiological functions remain unclear.

Recently, MARCH8 was identified as a novel antiviral factor against HIV-1. Tada et al. initially noticed that MARCH8-expressing lentiviral vectors had low infectivity and later found that a large amount of MARCH8 is specifically expressed in terminally differentiated myeloid cells, such as macrophages and dendritic cells. Ectopic expression of MARCH8 in virus-producing cells did not affect viral production but significantly decreased viral infectivity. MARCH8 was demonstrated to drastically reduce HIV-1 virion incorporation of envelope glycoproteins by downregulating it from the cell surface, possibly through their interaction, resulting in a significant reduction of viral infectivity (Fig. 1a). The same inhibitory effect was observed in virions containing envelope proteins from HIV-2 (human immunodeficiency virus-2), SIV (simian immunodeficiency virus), MLV (murine leukemia retrovirus), or vesicular stomatitis virus (VSV). Intriguingly, VSV G-glycoprotein was more sensitive to the inhibitory effect of MARCH8, indicating a wide-spectrum inhibition of enveloped viruses by MARCH8. Importantly, the endogenous MARCH8 was highly expressed in monocyte-derived macrophages and dendritic cells, and the depletion of MARCH8 in macrophages remarkably increased the infectivity of virions generated from these cells [19]. Their data, therefore, suggested that higher expression of MARCH8 in terminally differentiated myeloid cells is a potent antiviral host transmembrane protein that reduces virion incorporation of viral envelope glycoproteins.

Interestingly, although MARCH8 was suggested to interact with HIV-1 Env, leading to its downregulation from the producer cell surface, neither HIV-1 Vpr, Vpu, nor Nef has detectable anti-MARCH8 activity, suggesting that HIV-1 lacks a mechanism to antagonize MARCH8 antiviral activity directly. HIV, especially HIV-1, may have evolved to benefit from host restriction protein MARCH8. The suppression of infectivity by MARCH8 and other effects likely leads to a small amount of replication of HIV in macrophages, resulting in minimal cellular damage. Furthermore, the virus could escape from the host immune system and permit its survival in the host [20].

The latest study from Tokunaga's lab showed that MARCH8 prevented viral infections by two different mechanisms. Pseudotyped VSV-G viruses, in which cytoplasmic lysine residues were mutated, were insensitive to the inhibitory effect of MARCH8, although those with a similar lysine mutant of HIV-1 Env remained sensitive to it. Indeed, the wild-type VSV-G, but not its lysine mutant, was ubiquitinated by MARCH8. Besides, the MARCH8 mutant, with a disrupted cytoplasmic tyrosine motif essential for intracellular protein sorting, did not inhibit HIV-1 Env-mediated infection while still affecting VSV-G-pseudotyped virus infection. The underlying mechanism is that MARCH8 decreases viral infectivity by downregulating envelope glycoproteins through two separate mechanisms mediated by a ubiquitination-dependent or tyrosine motif-dependent pathway [21]. Further investigations are required to clarify the more comprehensive host defense mechanisms for MRACH8.

Except for inhibiting HIV's envelope glycoprotein, MARCH8 also blocks Ebola virus (EBOV) glycoprotein (GP) incorporation via surface downregulation. The underlying mechanism is that MARCH8 interacted with EBOV GP and furin and preserved Golgi's GP/furin complex. MARCH8 did not decrease the GP expression or affect the GP post-translation modification by high-mannose N-glycans in the endoplasmic reticulum (ER), but it inhibited the development of complex N-glycans on the GP in the Golgi (Fig. 1c). Besides, the GP O-glycosylation and furin-mediated proteolytic cleavage were inhibited. MARCH8 also blocked the furin-mediated cleavage of HIV-1 Env (gp160) and the highly pathogenic avian influenza virus H5N1 hemagglutinin (HA) [22]. All these data indicate that MARCH8 has a very broad antiviral activity by preventing different viral fusion proteins from glycosylation and proteolytic cleavage in the Golgi, which restrains their translocation from the Golgi to the plasma membrane and incorporation into virions.

MARCHF8 is also shown to suppresses porcine epidemic diarrhea virus (PEDV) replication by targeting and degrading virus nucleocapsid protein with selective autophagy [23]. Mechanically, MARCHF8 is recruited by BST2 to catalyze the ubiquitination of the PEDV N protein. The ubiquitinated N protein is then recognized by cargo receptor CALCOCO2/NDP52, which delivers it to autolysosome for degradation via the selective autophagy pathway (Fig. 1a).

### Proviral MARCH proteins

**Fig. 2:**
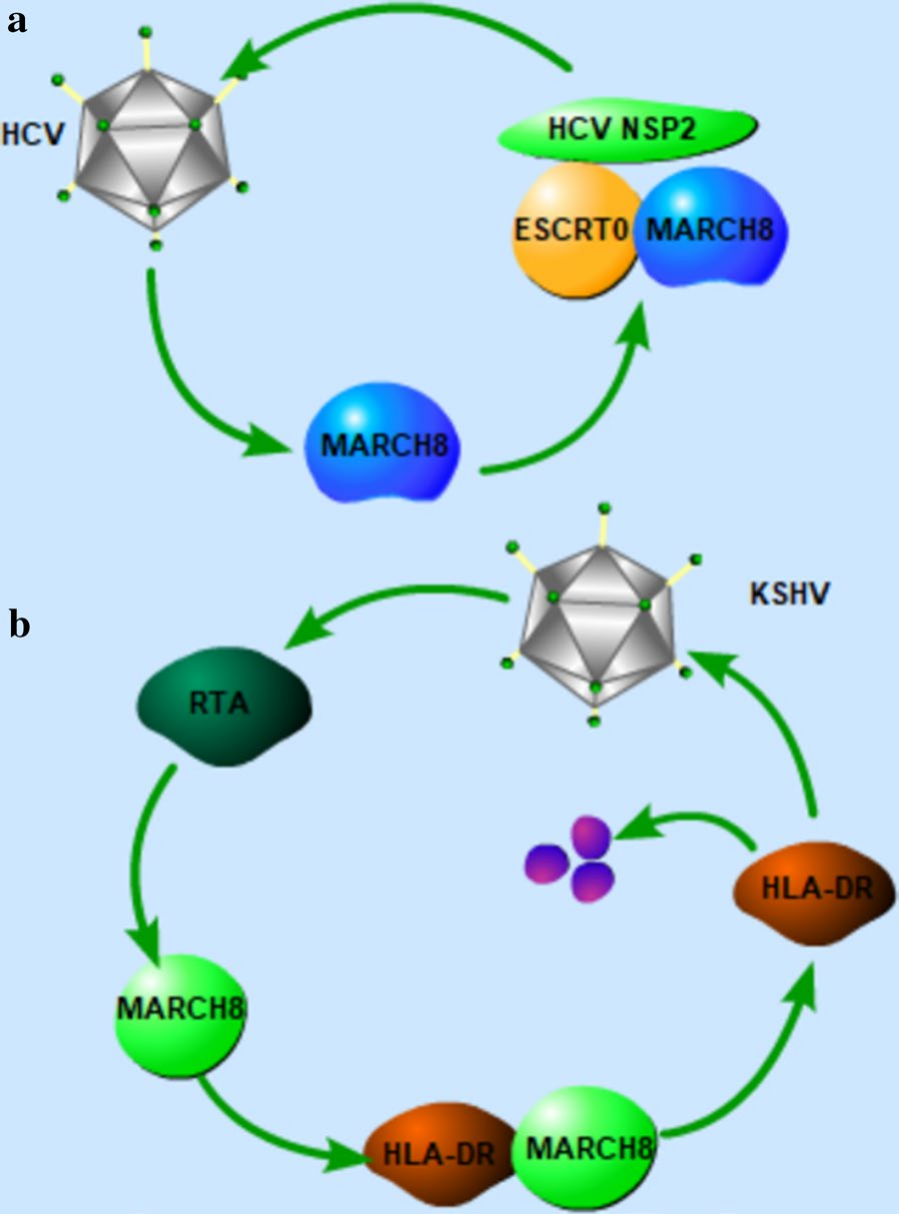
The proviral functions of MARCH family proteins. **a** HCV infection upregulates MARCH8 expression, and MARCH8 ubiquitinates the HCV NSP2 to promote the viral envelopment. **b** KSHV RTA promotes MARCH8 expression, and MARCH8 targets HLA-DRα for degradation and finally promotes viral replication. HCV NSP2: hepatitis C virus nonstructural protein 2

Except for MARCH8 as an antiviral factor, MARCH8 is also a proviral factor. MARCH8 ubiquitinates the hepatitis C virus (HCV) nonstructural protein 2, mediates viral envelopment, and plays a critical role in HCV infection [24]. The underlying mechanism is that MARCH8 mediates K63-linked ubiquitination of HCV NS2 and ESCRT recruitment (Fig. 2a). MARCH8 is essential for HCV, dengue, and Zika viruses' infection and specifically mediates HCV envelopment, indicating that MARCH8 is a potential target for host-targeted antiviral strategies.

Interestingly, Kaposi's sarcoma-associated herpesvirus (KSHV) replication and transcription activator actively upregulate the transcription of MARCH8 and induces its cellular expression to downregulate human leucocyte antigen DRα, a member of the major histocompatibility complex class II molecules (Fig. 2b), indicating that KSHV manipulates host MARCH8 to promote its immune evasion [25].

## Conclusion

In summary, MARCH E3 ligase family have now emerged as potent modulators and play a dual role in viral infections: for one hand, they contribute to the cellular antiviral functions by disrupting viral production, but the same MARCH ligases can be repurposed by other viruses to enhance their infectivity, or to downregulate important immunoreceptors to dampen the immune responses and help viral replications [26]. The recently identified MARCH family functions in antiviral defense and its proviral roles highlight the need to expand our understanding of MARCH biology to take advantage of MARCH ligases to enhance their antiviral effect and develop new antiviral therapy.

## Data Availability

Not applicable.

## Abbreviations

RING-CH: Really interesting new gene C4HC3 cysteine-histidine
MARCH: Membrane-associated RING-CH
HIV-1: Human immune deficiency virus
HBV: Hepatitis B virus
HCV: Hepatitis C virus
HCC: Hepatocellular carcinoma
HBX: Hepatitis B viral x
PEDV: Porcine epidemic diarrhea virus
KSHV: Kaposi's sarcoma-associated herpesvirus
MHC: Major histocompatibility complex
IL: Interleukin
TRAIL: TNF-related apoptosis-inducing ligand
HIV-2: Human immunodeficiency virus-2
SIV: Simian immunodeficiency virus
MLV: Murine leukemia retrovirus
VSV: Vesicular stomatitis virus
EBOV: Ebola virus
ER: Endoplasmic reticulum
HA: Hemagglutinin
